# The relationship between sports performance, physical activity and e-cigarette use among Australian adolescents: A qualitative study

**DOI:** 10.18332/tid/199474

**Published:** 2025-03-02

**Authors:** Amelia Yazidjoglou, Christina Watts, Grace Joshy, Emily Banks, Becky Freeman

**Affiliations:** 1 National Centre for Epidemiology and Population Health, The Australian National University, Canberra, Australia; 2 The Daffodil Centre, Woolloomooloo, Australia; 3 School of Public Health, Faculty of Medicine and Health, The University of Sydney, Sydney, Australia

**Keywords:** electronic cigarettes, sports performance, fitness, health effects, adolescents

## Abstract

**INTRODUCTION:**

In 2022–2023, 30% of Australian adolescents aged 12–17 years had used electronic cigarettes (e-cigarettes). Adolescents are particularly vulnerable to the negative health impacts of e-cigarettes. Although sport and physical activity participation have been postulated as potential protective factors against e-cigarette use, evidence on their relationship is limited and no qualitative data are available. This study aims to qualitatively explore the relationship of e-cigarette use, sport participation and physical activity, among Australian adolescents aged 14–17 years.

**METHODS:**

A total of 96 participants aged 14–17 years completed 78 online single or paired semi-structured qualitative interviews, as part of the Generation Vape project, during February–May 2023. All discussion was unprompted. Reflexive thematic analysis was applied and inductive coding undertaken.

**RESULTS:**

Of the 96 participants, 52 (54%) volunteered data relating to this topic. Sport participation and athletic performance were perceived as key drivers of protective adolescent e-cigarette use behaviors including abstinence, cessation and ‘responsible use’. Most current and former users reported experiencing health harms attributed to e-cigarettes – predominantly shortness of breath – during day-to-day physical activity such as walking or climbing the stairs and during sporting activities. Some users reported no difference in fitness attributable to e-cigarette use and former users reported improvements after quitting.

**CONCLUSIONS:**

Sport participation was considered important by adolescents and related it to e-cigarette patterns of use; and e-cigarette users described experiencing negative health effects in sport and fitness settings. E-cigarette use in adolescents may jeopardize the physical, mental and social benefits of engaging in sport and recreational physical activity. There is a need for greater regulation of e-cigarette industry sponsorship of sport to reduce adolescent exposure to e-cigarette marketing and promotion. E-cigarette prevention campaigns that highlight impacts on sport maybe an effective intervention to support overall adolescent wellbeing.

## INTRODUCTION

In a 2022–2023 national survey, 30% of Australian adolescents aged 12–17 years reported ever having used electronic cigarettes (e-cigarettes)^1^, despite national laws and policy that prohibit the sale or supply of e-cigarettes to those aged <18 years^2^. Many products are specifically designed and marketed towards adolescents^3^. Numerous e-cigarette health impacts have been identified including: addiction; poisonings both intentional and accidental; nicotine toxicity from inhalation; burns and injuries; lung injury; and increased smoking uptake in non-smokers^4-6^. The adolescent brain undergoes extensive neural development making adolescents particularly vulnerable to both the short- and long-term health impacts of nicotine^7^. Given the harms of e-cigarettes and the high prevalence of use, it is crucial to maximize efforts that prevent e-cigarette use among adolescents.

Sport participation and exercise are often postulated as potential protective factors against some risk behaviors, such as smoking, as they support physical health and promote overall wellbeing, encourage self-discipline and foster success-oriented mindsets, and behaviors that could compromise these benefits should be avoided^8^. In Australia in 2019, 89% of adolescents aged 15–17 years participated at least once a week in physical activity for sport, exercise or physical recreation. Fitness/gym, athletics and football/soccer were the most popular, and participation was motivated by fun/enjoyment in 71% and by health reasons in 64%^9^. Several studies have suggested that active adolescents are less likely to be regular smokers while others report no association or that physical activity or sport participation is associated with greater odds of tobacco smoking with variation by the frequency and intensity of physical activity, the competitive level and type of sport, and age and sex^10^. For example, one study found that compared to adolescents participating in sprint and resistance sports, those in team sports were three times more likely to be current smokers and another found that adolescents had four times the odds of smoking progressions (initiation or increased use) for every 30 min/week spent engaging in activities such as sport fighting, competitive wrestling and cycling, while those participating in racquet sports and swimming had around half the odds of smoking progression compared to non-participants^11-13^. Internationally, few studies have examined the relationship between adolescent e-cigarette use and physical activity, and we were not able to locate any that had done so qualitatively.

One cohort study found current e-cigarette users were more likely than non-users to participate in any type of physical activity^14^, one found that team sport, but not individual sport participation, was associated with greater odds of e-cigarette experimentation but among boys only^15^, and another cohort study found adolescents who met the physical activity guidelines were more likely than other adolescents to initiate e-cigarette use^16^. From cross-sectional data, compared to non-users, e-cigarette users were more likely to engage in various types of physical activities or sport, particularly for males^17^ and e-cigarette users were less likely to obtain ≥60 min/day moderate-vigorous physical activity but were no different for other physical activities^18^. Also five cross-sectional studies explored how sports participation related to e-cigarette use, finding that: competitive sport, sport outside of school and 60 min/day moderate-vigorous physical activity were positively associated with e-cigarette use^19^; any team sport participation was associated with use^20^; participation was associated with lower odds of use^21^; there was no difference in use by sport participation^22,23^.

The quantitative evidence – which is largely cross-sectional – on the interaction between sport, physical activity and e-cigarettes among adolescents is limited with inferences on causality and directionally unavailable and there is an absence of data describing potential mechanisms driving the relationship. It is unclear whether e-cigarette use influences physical activity participation with implications for adolescent health more broadly or supports sport participation as a protective factor against e-cigarette use, and, in fact, it may suggest the opposite. Therefore, there is a need to explore this relationship in more depth to better understand its complexity and nuance. To our knowledge, this is the first study aiming to qualitatively explore the relationship of e-cigarette use to sport participation and physical activity, including the impacts of sport on e-cigarette use and vice versa, and is based on data from adolescents aged 14–17 years.

## METHODS

This study used online single or paired semi-structured interviews from the Generation Vape research project – a four-year (2021–2025) multi-wave study with six-monthly quantitative and yearly qualitative data collection – designed to explore e-cigarette awareness, perceptions, attitudes, and knowledge among Australian adolescents aged 14–17 years^24^. Ethics approval for the project was granted by the University of Sydney Human Research Ethics Committee. A professional research recruitment agency used online panels for recruitment. Equal quotas of participants by e-cigarette use (ever and never users), sex (males and females), and school type (private and government) and 75% metropolitan participants (to reflect the geographical distribution of the general population) were obtained to enable the collection of a broad spectrum of experiences from participants with a range of different characteristics^24^. Respondents were screened following initial contact and were eligible for inclusion if they lived in Australia, had heard of e-cigarettes and reported that their age was 14–17 years. After receiving a Participant Information Statement, consent was obtained from both parents and participants.

Interviews were conducted during February–May 2023 (wave four) and consisted of 78 interviews (60 single and 18 paired) including 96 participants. They were approximately 30 minutes in length and held via Zoom by one of two interviewers using a piloted discussion guide. Interviews were audio recorded, transcribed verbatim and de-identified. Participants were not directly asked about sport and e-cigarettes and there were no specific questions relating to this in the interview guide. All discussion was raised unprompted by the participants with possible probing questions by the interviewer for follow-up and greater understanding. Therefore, the analytical sample is derived from a subset of the 96 participants who, by their own account, volunteered information on this topic.

The reflexive thematic analysis process, as outlined in Braun and Clarke^25^, was adopted for data synthesis as it facilitates the development of patterns of meaning (themes) while acknowledging the active role of the researcher in analysis. The process involved several – not necessarily sequential – phases^25^. Familiarization of the dataset occurred via repeated listening of audio recordings and reading of transcripts. Manual inductive coding was undertaken and codes, associated text excerpts and participant characteristics were copied into an excel spreadsheet. Codes were generally semantic with latent or higher level interpretations recorded in an accompanying note (Supplementary file Table 1). Coding was completed by one reviewer (AY), then revised and discussed with all authors, from which initial themes were generated^25^. Themes were reviewed and refined – with the input from all authors – and data were arranged into a logical and meaningful narrative guided by the research aims.

## RESULTS

The relationship between sports, physical activity and e-cigarettes was important to adolescents, as was demonstrated by the wealth of discussion which was entirely unprompted and initiated by participants. Of the 96 participants in the sample, 52 (54%) discussed the subject in some manner [52% females; 65% 16–17 years; 56% government schools; 19% never users; 21% former users; and 60% ever users (current and occasional)].

### Influence of sport on e-cigarette use

Many adolescents greatly valued their ability to participate in sport, perceived that it was critical to their decisions regarding e-cigarette abstinence and cessation, and impelled them to implement strategies to lessen or mitigate e-cigarette harms. No participant initiated using e-cigarettes as a method of improving sports performance (Table 1).

**Table 1:** The influence of sport on e-cigarette use including abstinence, quitting and strategies to mitigate e-cigarette harms, interviews conducted February–May 2023, Australia

*Themes*	*Quotes*
**Sport participation and performance as a driver for non-use**	*‘Cause I do a lot of netball … and I’m trying to like work really hard to get like higher up on netball, like try out for state teams, so I haven’t really thought about vaping, cause I think that would probably impact on what I wanna do.’* (Never user, 14–15 years, female)
	*‘Cause I play basketball, like I train 3 times a week … so like if you vape it’s gonna damage your lungs, and it’s gonna affect you like, it’s not cool. And especially like a lot of people like play like footy and basketball, and soccer, and like if you’re vaping and you gotta run like a lot, like that’s not gonna help you. Like it’s only damaging you ... like you can tell if someone’s like they’re out of breath super easily because they do it a lot.’* (Never user, 14–15 years, male)
	*‘I like to I like to keep active, I like to play sports and like the side effect like the stuff that it does to your lungs, like how people are like struggling to breathe and after doing small activities is just not for me ... I like to stay active and I like to be healthy so that’s not something I would like to do.’* (Never user, 14–15 years, male)
**Sport participation as a driver for quitting**	*‘I started trying to get fitter as well, and vaping wasn’t helping, so that was another reason to [quit] … I just started working out and stuff, and I was thinking about doing boxing and AFL, but yeah … I can’t do that while vaping, cause that’s just it’s it you get the puffs so easy ... like gasping for air.’* (Former user, 14–15 years, male)
	*‘Yeah the nicotine. And you know you’re addicted when it’s starting to affect you. Like with the basketball. That’s when I knew I needed to stop.’* (Former user, 16–17 years, male)
**‘Responsible’ e-cigarette use to maintain sport participation and performance**	*‘Um, I reckon if I bought another one and kept buying them it would definitely have an effect over the years. But I think currently at the rate that I’m, really vaping which is always constant, it’s not having huge effects on my sport.’* (Current user, 16–17 years, male)
	*‘Because I have, I play pretty high-level sport, like I have sporting commitments. Like I try it, like every now and, now and then whatever you know? But I never continuously go on, and vape just because of the effects that it has on you.’* (Current user, 14–15 years, male)
	*‘There’s been times where like the first time I quit, was because I was so worried about losing my like ability to go for a run, or to go for a walk and feel ok. Because like I had very healthy friends who started vaping, and then all of a sudden like I was running faster than them, and it was just very weird. And also, I used to be very big on the gym, so I was worried that I wouldn’t be able to do like um like hit new PB’s or um just to hit bigger weights, so I just had to stop, because I was really worried about my body. But now, not so much, because I, I think I’m a little smarter about the way that I go about it. Um like I don’t vape nearly as much as I used to vape, at all. And I only vape when it’s necessary.’* (Current user, 16–17 years, female)
**Sport/physical activity protects against e-cigarette harms**	*‘If you stay active, and you’re always running or doing cardio and everything, it doesn’t really do much to you … It’s not that bad … so if you’re actually like, if you’re staying active, and everything, like that then it’s, it doesn’t harm you too much ... It cuts, cuts down the risk of, any long- or short-term things.’* (Current user, 14–15 years, male)

*Sport participation and performance as a driver for non-use*

Sports participation (participation of any level/type) was often raised as a reason for not initiating e-cigarette use among never users. While some adolescents framed their biggest concern as an inability to participate in sport or being physically active more generally, others considered the impacts on their sport performance (ability to perform well or be competitive) or potential for selection in higher levels of competition. Several participants identified the specific e-cigarette health impacts (generally breathlessness and lung damage) that they anticipated as most likely to impact their sport participation.

*Sport participation as a driver for quitting e-cigarettes*

For some participants, observing their own poor sports performance or decline in fitness and causally attributing this to their e-cigarette use, was a compelling signal that they needed to quit. A few participants spoke of e-cigarettes as a barrier or hindrance in achieving their sport and fitness goals, which prompted them to quit.

*‘Responsible’ e-cigarette use for maintaining sport participation and performance*

For some participants, their ability to participate and perform well in sport was held in such high esteem that they adapted their e-cigarette use behaviors – by abstaining from purchasing their own device or using less frequently and only when ‘necessary’ – to try and mitigate any potential impacts e-cigarettes might have. They evoked the idea that there may be a ‘responsible’ pattern of e-cigarette use for supporting continued sports performance.

*Sport participation protective against e-cigarette harms*

One participant (current e-cigarette user) remarked that the benefits of engaging in physical activity and maintaining fitness was such that it lessened any potential immediate or long-term e-cigarette harms that may be experienced.

### Influence of e-cigarette use on physical activity or sports performance

Many current and former e-cigarette users described experiencing adverse e-cigarette effects in relation to their general physical activity and sports performance. While respiratory effects, such as breathlessness, were commonly raised in relation to sport or fitness, other potentially relevant physical or mental health impacts, such as increased heart rate or anxiety, were not mentioned. No participant reported improved sporting ability associated with e-cigarette use (Table 2).

**Table 2:** The influence of e-cigarettes on physical activity or sports performance, interviews conducted February–May 2023, Australia

*Themes*	*Quotes*
**Shortness of breath**	*‘Being able to breathe. I’ve got rugby starting soon and, and like it’s hard to keep up.’* (Current user, 16–17 years, female)
	*‘Yeah, well you know like you always have like a shortness of breath, like um like even for me, I play lots of sports and whatnot, and um, but I definitely do like feel shortness of breath, and um that’s pretty annoying.’* (Current user, 16–17 years, male)
**Decline in lowintensity physical activity**	*‘I do know that it like exercise it slows down, like my exercise, and like I was going on a walk, I get really puffed easily … cause as I was on a walk, I took my dog for a walk, and like I was getting, like I felt really unfit.’* (Current user, 14–15 years, female)
	*‘I don’t think it really stops me from anything. It definitely makes me more out of breath … like whenever I’m like say cleaning my room or whatever, or like bending over or whatever, it kinda gets me out of breath … I don’t really like run much anymore.’* (Current user, 16–17 years, female)
	*‘… it’s actually making everyone worse. Cos like, I see some of my friends, two months ago they were like, completely fine with walking upstairs. But now that they’re constantly zeroing it, like I know it’s not good to vape, like normally ... Like they’re struggling to walk up the stairs.’* (Current user, 16–17 years, female)
	*‘I remember um I used to be able to like, walk and not be tired or run. Run up my road and be fine with it and then like over these couple of months of vaping, years, and now I struggle and … I’m not really that unfit like my whole job is to walk and, everything so I think it was just, vaping.’* (Ex-user, 16–17 years, female)
**Decline in sport performance or fitness**	*‘... also just whenever I would try and like do any sort of physical activity it would affect my breathing … my fitness was just, it was declining as I was progressively using it more, so it was affecting my breathing.’* (Ex-user, 16–17 years, male)
	*‘I play league tag … when, I mean before I …vaped, I would just feel like fit I would barely lose my breath like, I felt so much healthier and like, could just run forever and then, I started doing them and now I’m just so short of breath and like, just am nowhere near as, fit as I used to be because obviously of the vapes.’* (Current user, 16–17 years, female)
	*‘Um, just like I don’t wanna get to the point where, like I’m, I’m running up the stairs and I’m out of breath. It’s more like I relate it back to like a basketball game where I used to be able to run like 40 minutes straight. I, I would play the full game, I would be fine ... Now it’s like … I’m guessing it’s because of [vaping], that um, like I’m playing for about, 20 minutes and then, I, I just need like maybe a 5-minute break and then I can, run out the rest ... It’s cos I’m really puffed out.’* (Current user, 14–15 years, male)
**No impact on performance**	*‘One of my old friends he used to like vape a lot, pretty sure he still does but um, we were doing like a beep test at school and he still won the beep test. He was like, still fitter than like the whole class and he, and he was like, vaped a lot so, not sure how that works.’* (Ex-user, 16–17 years, male)
	*‘It doesn’t really affect me at the moment, like I haven’t seen any drop in, you know I can run, or how much exercise I can do.’* (Current user, 16–17 years, male)
**Improved performance once quit**	*‘I personally am a track athlete ... So, training, I think and the period of time where I was like regularly using a vape, it was very difficult, not very difficult, but I like just wasn’t running to full potential or standard without like, I dunno, loss of breath. So when I stopped, when I fully got rid of them, I like found myself, like I could actually do a lot of things easier.’* (Ex-user, 16–17 years, female)
	*‘My fitness is getting a lot better, which I like a lot, and um, it’s just not good for ya, there’s no point restarting.’* (Ex-user, 14–15 years, male)

*Shortness of breath*

Breathlessness, struggling to breathe and being puffed were consistently and frequently reported by participants in relation to their general fitness or sports performance. This was the most overt and widely attributed direct impact of e-cigarettes.

*Decline in low-intensity physical activity*

Current and former users reported feeling the effects of e-cigarettes while engaging in day-to-day activities or low-intensity activities. For example, activities such as walking or climbing stairs were identified as more difficult since they started vaping. One participant seemed unconcerned when describing being out of breath from bending over while cleaning their room.

*Decline in sports performance or fitness*

Participants reported noticeable decline in their ability to perform more strenuous activities with many current and former users, describing how the breathlessness experienced as a result of vaping directly interfered with their sports performance. Many spoke of general health and fitness decline in the context of sport while others identified specific activities that were impacted such as running or basketball. For one participant, the decline in performance had begun to actively exclude him from participating in his sport for temporary intervals as he now required more breaks.

*No impacts on sports performance*

A few participants had not personally experienced any decline in sports performance. Also, no apparent decline in fitness was witnessed in some peers who were e-cigarette users engaged in athletically demanding activities.

*Improved performance after quitting e-cigarettes*

Former e-cigarette users described noticeable improvement in their fitness after quitting, with activities becoming easier. One participant’s experience of the benefits from quitting was so influential it was identified as a motivator for preventing relapse.

## DISCUSSION

This study demonstrates the complex and dynamic relationship between sport, physical activity and e-cigarette use, and how this is perceived among Australian adolescents. Discussion of the relationship between sport and e-cigarettes was unprompted, but was repeatedly raised by participants signifying the salience of the issue for adolescents. Sport participation and performance was viewed of great importance with its preservation driving abstinence (non-use) and cessation. For one participant, the benefits from sport were considered so impactful that they negated any potential harms of e-cigarettes. For most adolescents, sport, fitness and physical activity were perceived to be negatively impacted by their e-cigarette use. Shortness of breath was the most common impact reported, which was even experienced during low intensity day-to-day activities such as walking or climbing stairs, although some did not report any impacts at all.

The current evidence regarding the relationship between sport and e-cigarette use is scarce, varied and quantitative. It broadly suggests that among adolescents, e-cigarette use is associated with greater physical activity levels and sports participation. Evidence from a Canadian prospective cohort study found that, compared to non-users, there was a greater likelihood of participating in moderate-vigorous physical activity, strengthening exercises and sport participation among current e-cigarette users^14^ with the findings supported by another Canadian study (cross sectional)^17^. However, cross-sectional data from the US found that there was no difference in the odds of vigorous physical activity or muscle strengthening physical activity between e-cigarette users and non-users, and that e-cigarette users were less likely to obtain ≥60 min/day moderate-vigorous physical activity^18^.

Evidence also suggests that sports participation is associated with greater e-cigarette initiation^16^, but may vary by gender, sport type and competitive level^15,19,20^. For example, cross-sectional data form the US found that team sport, but not individual sport participation, was associated with greater odds of e-cigarette experimentation among boys but not girls^15^, and that any team sport participation was associated with e-cigarette use, and was greater for intermittent users than frequent users^20^. Among Canadian adolescents, competitive sport, sport outside of school and 60 min/day moderate-vigorous physical activity were positively associated with e-cigarette use while competitive sport was negatively associated^19^. However, other cross sectional evidence found sports participation was associated with lower odds of e-cigarette use among Irish adolscents^21^; that there was no association between e-cigarette use and intramural sport participation in Canadian adolscensts^22^, and by competitive sport participation (frequency or type) among US adolescents^23^. While this evidence would appear counter to our findings, most data are cross-sectional and describe only the relationship at a single point in time; however, it is unlikely that this relationship remains static over time. Instead, the influence of sport on e-cigarette use or vice versa, is dynamic and as evidenced by our study, it changes depending on efforts to improve sporting performance, and personal experiences of adverse e-cigarette health effects or decline in sporting participation and fitness. Given the importance of the sport–e-cigarette interaction for adolescents and the fluidity of the relationship, a valuable opportunity presents for leveraging this interaction to promote e-cigarette prevention campaigns to support overall adolescent wellbeing.

Although there is conclusive evidence that use of e-cigarettes can lead to e-cigarette or vaping associated lung injury (EVALI), there is insufficient evidence regarding other important clinical respiratory outcomes (such as chronic obstructive pulmonary disease), and subclinical outcomes including lung function and lung capacity^6^. One small experimental study was identified that considered cardiorespiratory impacts of e-cigarettes in young adults, which found significantly reduced cardiorespiratory fitness among e-cigarette users compared to non-users^26^. Although certain kinds of evidence may be lacking, this study shows that reductions in lung function – perceived or otherwise – and shortness of breath are meaningful and important to adolescents. These impacts are considered so detrimental, particularly to their sporting aspirations or overall fitness, that they are reported as contributing to patterns of use including abstinence and cessation. While these symptoms are indicators of respiratory function, potentially short-lasting and may not be indicative of clinical disease, given their salience for adolescents and the very real negative impacts experienced, it is important that they are not overlooked in health messaging. An Australian anti-vaping campaign ‘Every vape is a hit to your health’ targeting participants aged 14–24 years has recognized the relevance of this symptom and made one of their key messages about breathlessness^27^. Made apparent through sport and physical activity, Australian adolescents report experiencing the health effects of e-cigarettes and these narratives, told in their own voice, provide compelling and believable dialogue that is likely to resonate among other adolescents. This juncture between sport, health and e-cigarettes likely provides another entry point for health promotion and e-cigarette prevention activities.

Aside from the physical benefits derived from physical activity and sport, there are numerous other benefits including supporting social, academic, and emotional wellbeing^28,29^. Sport participation and recreational or leisure time physical activity can support mental health, foster community connections, and promote social interactions and relationships^29,30^. There is also evidence suggesting that children and adolescents that participate in sport are more likely to be active in adulthood than those that do not^29^. If the effects of e-cigarettes experienced by adolescents, such as shortness of breath, reduce their ability to engage in sport or recreational physical activity, adolescents miss out on the complete and extensive range of benefits it provides.

The public health implications of the relationship between sport and e-cigarette promotion must also be recognized. Elite athletes are often positioned as positive role models for adolescents, promoting not only athletic prowess but also healthy lifestyles. It can be particularly damaging if these public personas either intentionally or unintentionally demonstrate favorable attitudes towards e-cigarettes that normalize their use or minimize their harms. For example, an Australian rugby player, subsequently dismissed over this incident, filmed and uploaded to social media a video of themselves vaping during half-time of a national league game^31^. In 2022, an e-cigarette retailer in the UK – with links to Phillip Morris International – recruited a former football star as campaign ambassador promoting vaping as the answer for achieving smoke-free societies^32^. Tobacco companies have a long history of sponsoring sporting players, teams and events as they aid in associating their brand with healthy lifestyles, reaching younger audiences and often provide an avenue to evade advertising restrictions^33,34^. Furthermore, the broadcasting of sporting events and the creation of programs centered on specific sports, such as Netflix’s ‘Drive to Survive’, the tobacco industry benefits from extensive brand exposure, with approximately 33% of the show ‘Drive to Survive’ displaying tobacco branding – predominantly e-cigarette and nicotine pouch products – equating to around 35 minutes per viewer^35^. Unsurprisingly, the e-cigarette industry has also adopted this tactic sponsoring football teams^32^, and sports grounds (Cigg-e Stadium in Wales, 2013)^36^. In recognition of the invaluable reach and access sport sponsorship provides to industry, policymakers should apply comprehensive bans on e-cigarette advertising and promotion, as a key component of effective tobacco control.

### Strengths and limitations

To the best of our knowledge, this is the first study to qualitatively investigate the relationship between sports, physical activity and e-cigarettes among adolescents. It explores in depth the many facets of the relationship and provides insight into the cyclical nature of sports and e-cigarettes from the adolescents’ perspective. While there was a wealth of data derived from unprompted discussion on sports and e-cigarettes, some topics or details may have been missed as the interviews were not specifically designed to examine the relationship between sports, physical activity and e-cigarettes. Online panels were used for recruitment, and although this is standard practice in qualitative research, it may have resulted in limited recruitment from among certain members of the community.

## CONCLUSIONS

Sport participation and performance were considered key drivers of adolescent e-cigarette use behaviors including abstinence, cessation and ‘responsible use’. Most current and former users reported experiencing health harms attributed to e-cigarettes – predominantly shortness of breath – during day-to-day activities such as walking or climbing the stairs and during sporting activities. E-cigarette use in adolescents may jeopardize the physical, mental and social benefits of engaging in sport and recreational physical activity. There is a need for greater regulation of e-cigarette industry sponsorship of sport to reduce adolescent exposure to e-cigarette marketing and promotion. Anti-vaping campaigns aimed at adolescents that stress the impacts on physical fitness are also a potential effective intervention.

## Supplementary Material



## Data Availability

The data supporting this research cannot be made available for privacy or other reasons.
