# Supplementary material for: The relationship between sports performance, physical activity and e-cigarette use among Australian adolescents: A qualitative study
Source: Tob Induc Dis. 2025 Mar 2;23:10.18332/tid/199474. doi: 10.18332/tid/199474 (PMC11871528; doi:10.18332/tid/199474)
Supplement: Supplementary file 1 [file TID-23-23-s1.pdf]

Supplementary Table 1. Codes and initial themes examples

Participant characteristics	Quote	Codes	Potential themes	Notes
Never user; 14-15 years; female	Cause I do a lot of netball… and I'm trying to like work really hard to get like higher up on netball, like try out for state teams, so I haven't really thought about vaping, cause I think that would probably impact on what I wanna do	- Athletic/sports performance gains key<br>- Perceived negative vaping impact on fitness<br>- Vaping not compatible with sporting goals<br>- Sport driving vaping abstinence	- Sport pivotal and is influencing vaping behaviour (including abstinence)	- Sport influence vaping
Current user; 16-17 years; female	There's been times where like the first time I quit, was because I was so worried about losing my like ability to go for a run, or to go for a walk and feel ok. Because like I had very healthy friends who started vaping, and then all of a sudden like I was running faster then them, and it was just very weird. And also, I used to be very big on the gym, so I was worried that I wouldn't be able to do like um like hit new PB's or um just to hit bigger weights, so I just had to stop, because I was really worried about my body. But now, not so much, because I, I think I'm a little smarter about the way that I go about it. Um like I don't vape nearly as much as I used to vape, at all. And I only vape when it's necessary	- Athletic/sports performance gains key<br>- Perceived negative vaping impact on fitness<br>- Vaping negatively impacting others fitness<br>- Quit for fitness reasons<br>- Moderate vaping to maintain fitness<br>- "Responsible" vaping	- Sport pivotal and is influencing or modifying vaping behaviour<br>- "Responsible" vaping	- Sport influencing vaping<br>- Sport driver of previous cessation but no longer seen as barrier<br>- Perception that vaping harms can be controlled for or mitigated

Participant characteristics	Quote	Codes	Potential themes	Notes
Current user; 14-15 years; female	I do know that it like exercise it slows down, like my exercise, and like I was going on a walk, I get really puffed easily… cause as I was on a walk, I took my dog for a walk, and like I was getting, like I felt really unfit	- Declines in low intensity physical activity<br>- Negative impacts of vaping on fitness<br>- Puffed/out of breath/breathlessness	- Declines in day-to-day fitness<br>- Declines in low intensity physical activity	- Vaping influencing fitness

© 2025 Yazidjoglou A. et al.
